# Importance of integrity of cell-cell junctions for the mechanics of confluent MDCK II cells

**DOI:** 10.1038/s41598-018-32421-2

**Published:** 2018-09-20

**Authors:** Bastian Rouven Brückner, Andreas Janshoff

**Affiliations:** University of Goettingen, Institute of Physical Chemistry, Tammannstr. 6, 37077 Goettingen, Germany

## Abstract

Intercellular junctions are important mechanical couplers between cells in epithelial layers providing adhesion and intercellular communication. Regulation of the junctions occurs in cellular processes such as layer formation, epithelial-to-mesenchymal transition, embryogenesis, and cancer progression. Many studies addressed the role of force generation in cells for establishing lateral cell-cell junctions and the role of cellular force transmission in tissue formation and maintenance. Our atomic force microscopy- (AFM) based study shed light on the role of both, tight junctions and adherens junctions for the mechanical properties of individual epithelial cells that are part of a confluent monolayer. We found that tight junctions are important for the establishment of a functional barrier-forming layer but impairing them does not reduce the mechanical integrity of cells. Depletion of ZO-1 results in a weak increase in cortical tension. An opposite effect was observed for disruption of E-cadherin-mediated adherens junctions using DTT. Opening of adherens junctions leads to substantial alterations of cellular mechanics such as reduced overall stiffness, but these changes turned out to be reversible after re-establishing disulfide bridges in E-cadherin by removal of DTT. We found that regulatory mechanisms exist that preserve mechanical integrity during recovery of disrupted adherens junctions.

## Introduction

Epithelial cells form a dense, stable cell layer lining the outer surface of tissue and organs. Mechanical strength and communication between the cells within a layer is provided by different cell adhesion sites including tight junctions (TJs)^1^, adherens junctions (AJs)^2^, desmosomes^3^, and gap junctions^4^. Establishment of these intercellular junctions divides polarised cells in apical and basolateral divisions. Owing to their motility and dynamic conditions epithelia are capable of monitoring development^5^, tissue healing^6^, and cancer invasion^7^. To fulfil their role in force transmission between cells lateral cell-cell junctions are connected to the actin cytoskeleton.

Tight junctions are the uppermost cell-cell connection at the lateral cell membrane of polarised epithelia. They form a physical barrier to control the lateral flux of ions, macromolecules, pathogens, and other solutes within the paracellular pathway^8^. In addition, tight junctions are responsible for the separation of apical and basolateral membrane lipids and proteins^9,10^. TJs consist of transmembrane proteins such as occludin, claudins, or JAMs and peripheral membrane proteins from the zonula occludens (ZO) or cingulin family^8^. The actin cytoskeleton is connected to this junctional complex via these zonula occludens proteins, including ZO-1, ZO-2, and ZO-3. As a consequence, loss of ZO proteins influences the actomyosin cortex structure at the tight junctions, for instance, ZO-1/-2 double knock down led to accumulating actin structures as well as an enhanced myosin IIB level at the adherens junctions^11^. Actin becomes more clustered at the apical cell side^11^. It was also recently found that epithelial tension and effective viscosity are increased in ZO-1/-2 lacking cells^12^.

Adherens junctions form a strong intercellular connection and thus are important for the lateral cell layer integrity. For a long time, these junctions were considered to be the key players for force transmission through intercellular junctions. AJs are formed by transmembrane proteins from the cadherin group. This protein superfamily can be divided into two subgroups. Approximately 20 subtypes of cadherins can be found in vertebrates (“classic” cadherins), among them epithelial cadherin (E-cadherin) and neural cadherin (N-cadherin). The extracellular amino-terminal EC1 domain of one cadherin binds to the same cadherin of an adjacent cell ending up in a homophilic *trans* dimer. This recognition is Ca^2+^-dependent^13^. At the innercellular membrane side, the cytoplasmic cadherin tail binds to p120 catenin, which is connected to *β*-catenin. *β*-catenin binds to *α*-catenin, which provides the linker to the C-terminal actin domain^14–16^.

While the molecular details of cell-cell junctions are well-known, their role for cellular mechanics on the mesoscale is largely unexplored. Trepat and co-workers recently demonstrated that the formation of epithelial cracks during cell sheet dilatation is caused by tissue stretching independent of epithelial tension resulting from a transient pressure build-up in the substrate during stretching and compression^17^. Essentially, the authors found that the cracks do not originate from detachment of the cortex from the membrane but are due to dissolution of adherens junctions. Even tight junctions remain mostly intact in these cracks.

Espinosa and co-workers used micropillar arrays and AFM indentation experiments on single cells and cell pairs to investigate the impact of the desmoplakin–intermediate filament linkage on cell mechanics^18^. They found that desmosome–intermediate filament networks are important contributors to cell stiffness in cell pairs and sheets underlining the importance of cell-cell contacts for cellular mechanics. Therefore, depending on the cell type different mechanisms might exist to regulate tension.

In the present study, we examined how epithelial cells regulate their mechanical behaviour when cell-cell junctions are impaired. We employed atomic force microscopy (AFM) which is proven to be a versatile tool not only for imaging but also to assess local mechanical properties of adherent cells^19,20^. Local mechanical properties are an important readout how cells respond to environmental changes^21–24^. We stopped tight junction formation by ZO-1 silencing and interfered with the integrity of adherens junctions by a chemical stimulus. Mechanical measurements were analysed using a minimal mechanical model based on the assumption that the restoring force to deformation originates mainly from cortical tension. Our data indicate that the impact of tight junctions is of minor importance for the mechanics of epithelial cells from a monolayer, while their morphology is changed considerably. Loss of adherens junctions, however, changes tension and area compressibility significantly, while the morphology of the cell layer is only slightly affected. Therefore, our study suggests that TJs and AJs impact cellular mechanics in different, opposite ways highlighting their role in establishing a tight but also flexible barrier tissue.

## Results

### Tight junctions are responsible for a complete, dense cell monolayer

Tight junctions form the uppermost cell-cell connections in epithelial cells. This junctional complex consists of the transmembrane proteins occludin and various claudins. On the inner cellular site, it binds to zonula occludens, which establishes the contact to the F-actin cytoskeleton^8^. We used short interference RNA (siRNA) against zonula occudens-1 (ZO-1) in order to examine the role of tight junctions for the mechanical integrity of epithelial cells. We chose to knock out ZO-1 since it is known to bind to nearly all relevant proteins of the tight junctional complex, especially the perijunctional actomyosin network. It was also shown by Anderson and co-workers that depletion of ZO-1 preserves the transepithelial resistance but generates appreciable changes in cell morphology and organisation of the actomyosin cortex^9^.

**Figure 1:**
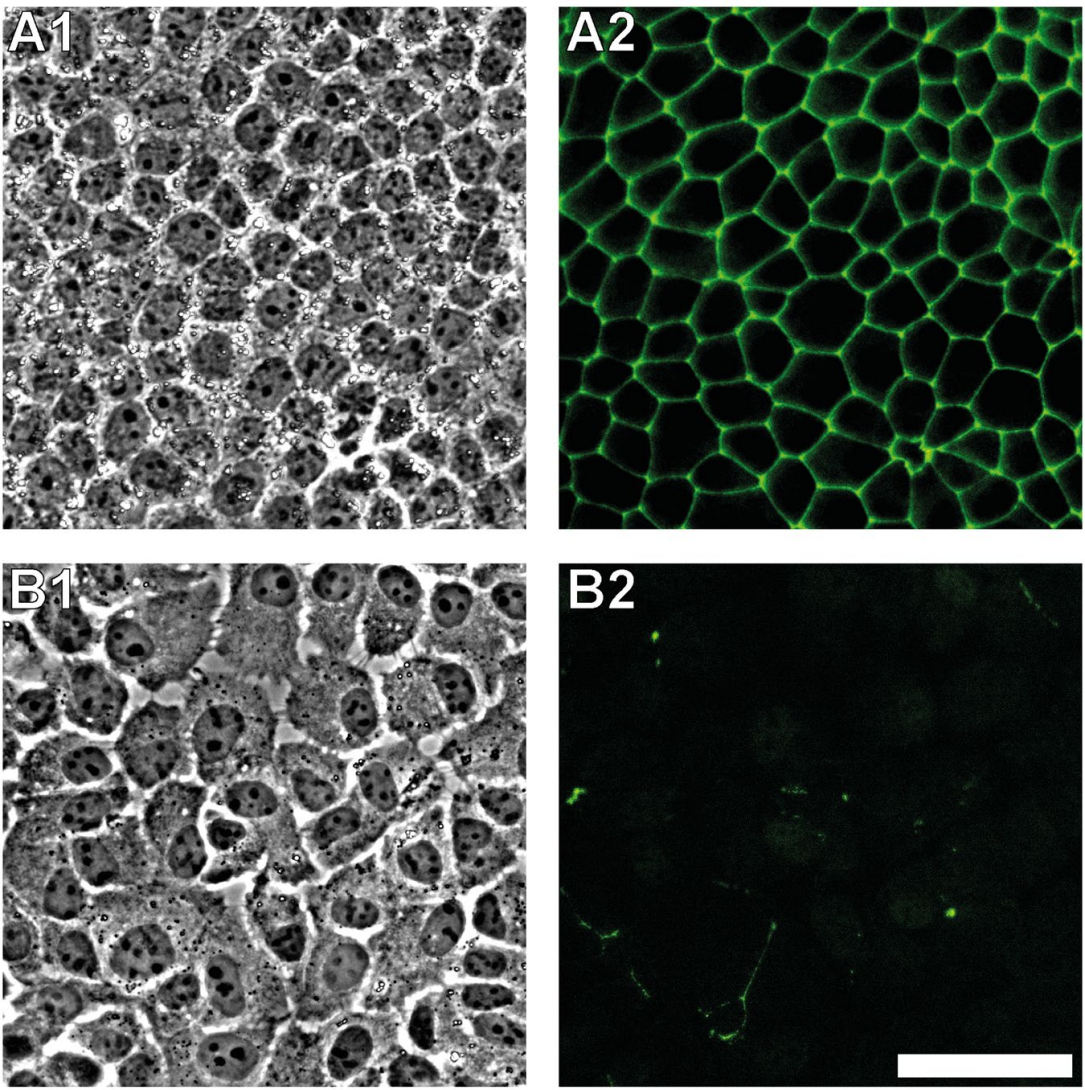
Transfection efficiency of siRNA against tight junction protein ZO-1. (**A**) Control cells. (**B**) Zonula occludens-1 silenced MDCK II cells. 1: Phase contrast images. 2: Fluorescence images for ZO-1. Scale bar: 50 µm.

**Figure 2:**
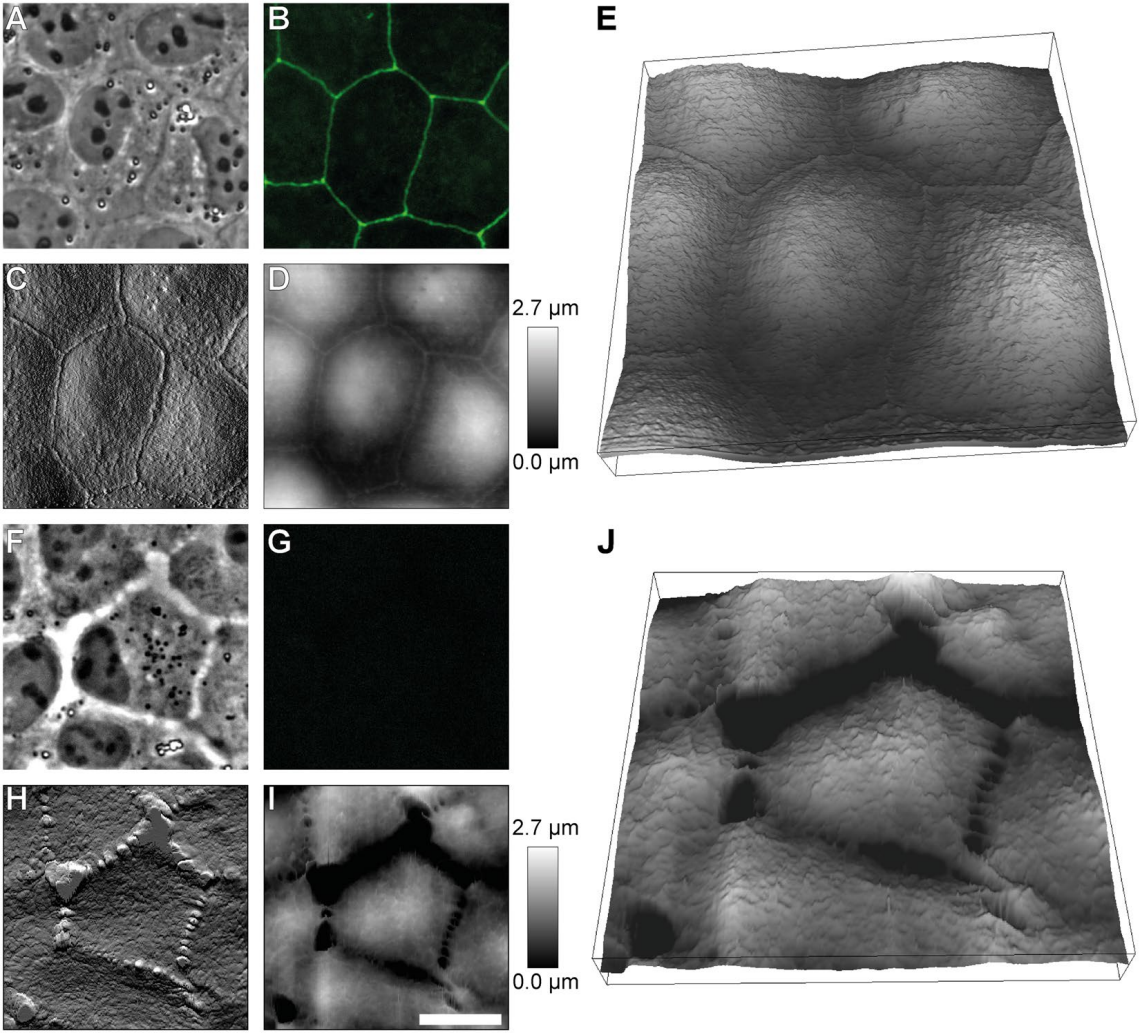
Topography and morphology of ZO-1 lacking MDCK II cells. (**A–E**) Untreated cells. (**F–J**) ZO-1 deficient cells. (**A**,**F**) Phase contrast images. (**B**,**G**) Fluorescence images for ZO-1. (**C,H**) AFM deflection images. (**D,I**) AFM height images. (**E**,**J**) Three-dimensional reconstructions of the same region shown in (**A–D**) and (**F–I**), respectively. Scale bar: 15 µm.

Phase contrast images of confluent MDCK II cells show a tight, densely packed cell monolayer (Fig. 1A1). Fluorescence images of ZO-1 stained cells show that tight junctions built up a gapless mesh surrounding each cell (Fig. 1A2). After applying siRNA against ZO-1 the cell layer still forms but is less uniform. Cells appear to be separated from each other and cell borders are brighter in phase contrast images (Fig. 1B1). ZO-1 is blocked with high efficiency by siRNA as nearly all cells lose their fluorescence signal (Fig. 1B2). This spatial separation between cells is further confirmed by AFM-imaging of ZO-1 deficient cells (Fig. 2). A continuous connection appears in AFM images for a fully established cell monolayer of untreated cells (Fig. 2A–E). In contrast, for ZO-1 deficient cells we found that adjacent cells are no longer connected to each other and only a few finger-like connections are visible (Fig. 2F–J).

**Figure 3:**
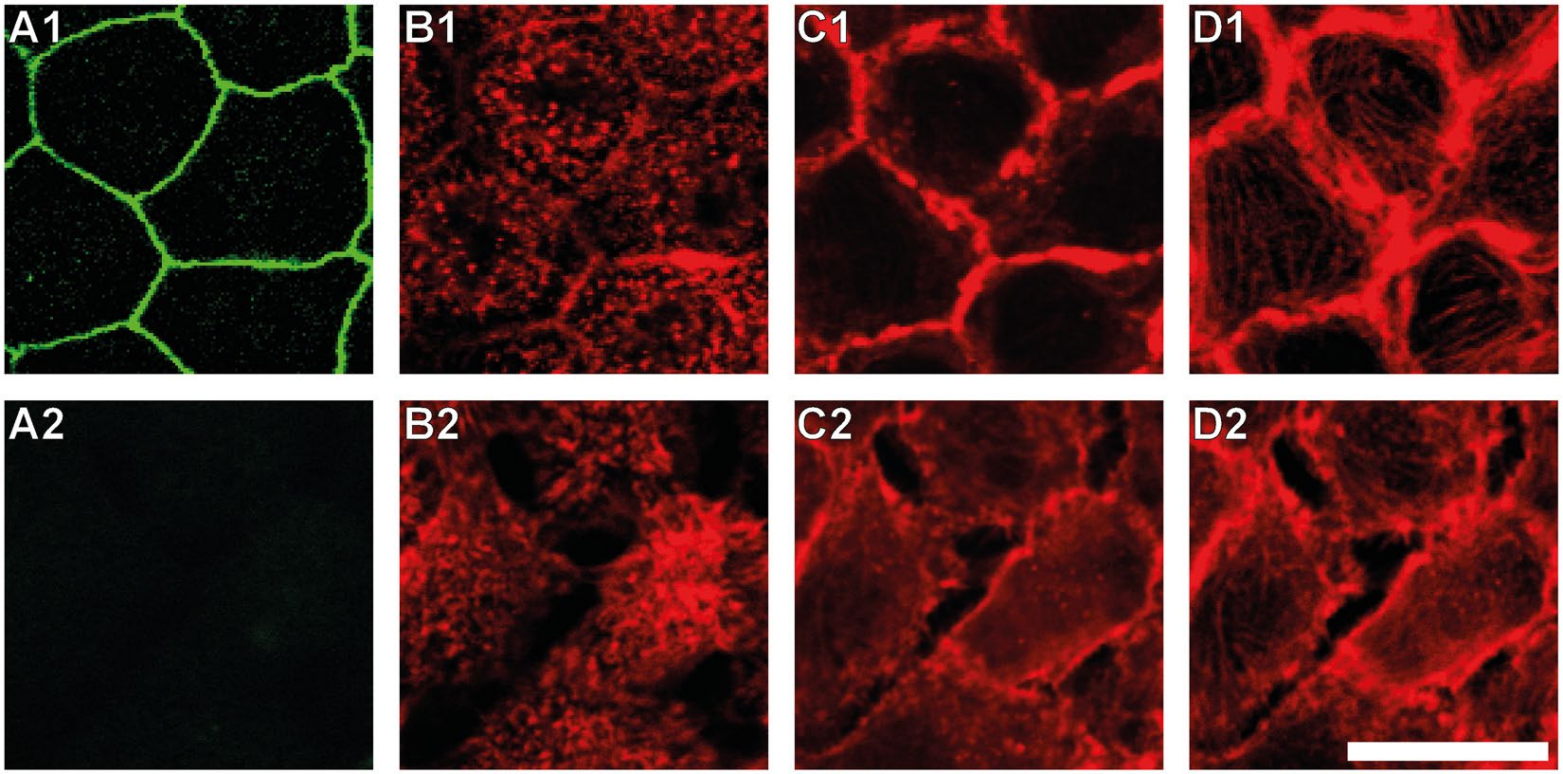
Changes in the F-actin distribution of ZO-1 lacking MDCK II cells. (**A**) Fluorescence images for ZO-1. (**B**–**D**) Fluorescence images for F-actin at the apical (**B**), central (**C**), and basal (**D**) focal plane. 1: Untreated cells. 2: ZO-1 silenced cells. Scale bar: 20 µm.

Since ZO-1 connects the tight junctional complex with the actin cytoskeleton, the actin distribution of cells after ZO-1 depletion was also analysed by fluorescence microscopy (Fig. 3). On the apical side small point-like structures are found for untreated cells (Fig. 3B1), reflecting the actin-filled microvilli of polarized MDCK II cells^25^. At the basal side stress fibres are visible (Fig. 3D1). For successfully ZO-1 silenced cells the actin distribution is more clustered on the apical side (Fig. 3B2), while on the basal side stress fibres disappear (Fig. 3D2). At the central focal plane actin fences untreated MDCK II cells, whereas a distinct cell-cell separation becomes visible for ZO-1 deficient cells and the actin signal appears blurry (Fig. 3C).

**Figure 4:**
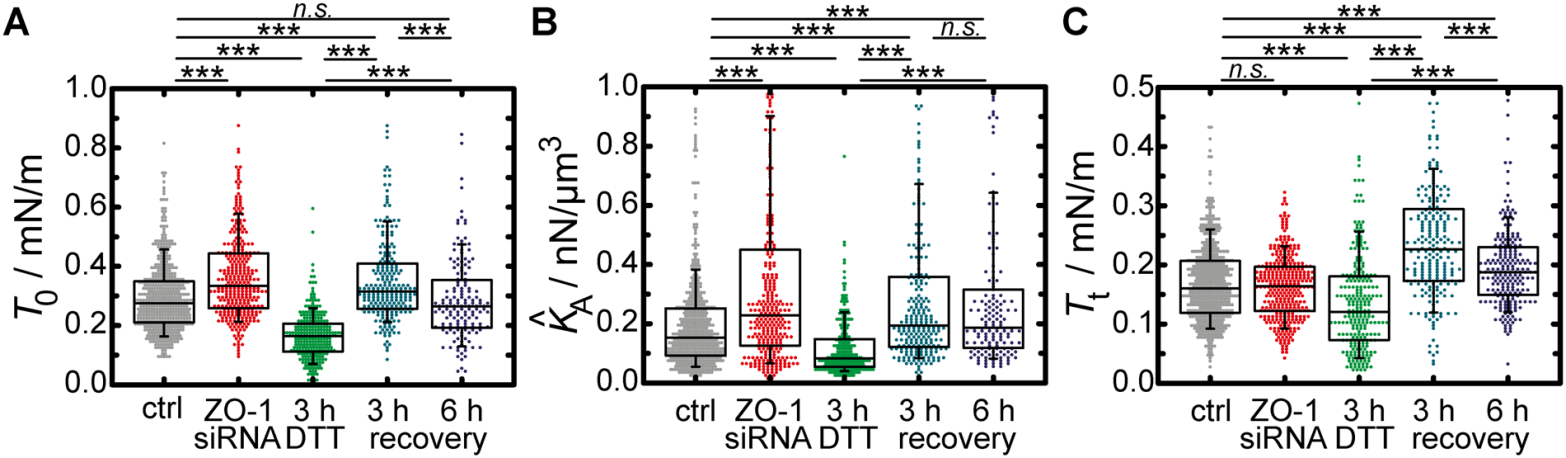
Impact of tight junctions and adherens junctions on the mechanical behaviour of MDCK II cells. (**A**) Overall tension comprising cortical tension and membrane tension. (**B**) Apparent area compressibility modulus. (**C**) Membrane tension. Box plots extend from the 25^th^ to 75^th^ percentile, whiskers from the 10^th^ to the 90^th^. Grey dots show data extracted from force-indentation curves recorded on untreated cells (ctrl), red ones were recorded on cells without zonula occludens (ZO-1 siRNA). Light green points represent data from cells exposed to 10 mm DTT for 3 h. Dark green and blue ones show mechanical parameters of cells where DTT was removed for 3 h and 6 h, respectively. (**A**,**B**) *n* = 501 (ctrl), 310 (ZO-1 siRNA), 275 (3 h DTT), 234 (3 h recovery), 151 (6 h recovery) analysed force-indentation curves. (**C**) *n* = 618 (ctrl), 337 (ZO-1 siRNA), 259 (3 h DTT), 219 (3 h recovery), 222 (6 h recovery) analysed force-retraction curves. A rank-sum test was performed to test the null hypothesis that the data of the indicated datasets are from populations with equal medians. Asterisks indicate that the null hypothesis was rejected at the 0.05% (***) significance level. *n*.*s*. indicates no significant difference at a 5.0% level.

Mechanical properties are inferred from AFM-based indentation experiments that provide information about cortical strength, membrane tension and excess membrane area. We indented siRNA treated cells with an AFM tip in order to analyse the impact of ZO-1 on these properties. Interestingly, although the cells are visibly separated from each other, the mechanical parameters are only slightly altered by ZO-1 depletion (Fig. 4). The biggest effect is an increased cortical strength after weakening of tight junctions. The average overall tension of untreated cells is found to be ${T}_{0}=(0.275\pm 0.010)\,\text{mN}/{\rm{m}}$ $({\rm{median}}\pm {\rm{SEM}})$. Cells with compromised tight junctions show a significantly increased overall tension of ${T}_{0}=(0.334\pm 0.009)\,\mathrm{mN}/{\rm{m}}$ (Fig. 4A). The apparent area compressibility modulus is also higher for cells lacking ZO-1 (${\hat{K}}_{{\rm{A}}}=(0.228\pm 0.007)\,{\mathrm{nN}/\mu {\rm{m}}}^{{\rm{3}}}$, control: ${\hat{K}}_{{\rm{A}}}=(0.153\pm 0.008)\,{\mathrm{nN}/\mu {\rm{m}}}^{{\rm{3}}}$) (Fig. 4B). The membrane tension *T*_t_, which is a measure for the strength of membrane-cytoskeleton attachment sites, is found to be $(0.161\pm 0.004)\,\mathrm{mN}/{\rm{m}}$ for untreated cells. Nearly the same value is found for ZO-1 lacking cells (${T}_{{\rm{t}}}=(0.164\pm 0.004)\,\mathrm{mN}/{\rm{m}}$) (Fig. 4C).

In order to exclude mechanical alterations due to the transfection procedure control measurements using non-targeting siRNA are shown in the supplementary information (suppl. Fig. S1)^26^. Applying the transfection protocol shifts mechanical parameters to slightly lower values, i.e. in the opposite direction.

In summary, ZO-1 is extremely important for the formation of a dense cell layer, which shields underlying cells. The morphology of ZO-1 lacking cells is changed tremendously but the overall stiffness of the cells increases only slightly compared to well-connected cells.

### Adherens junctions influence topography and mechanics of cells

**Figure 5:**
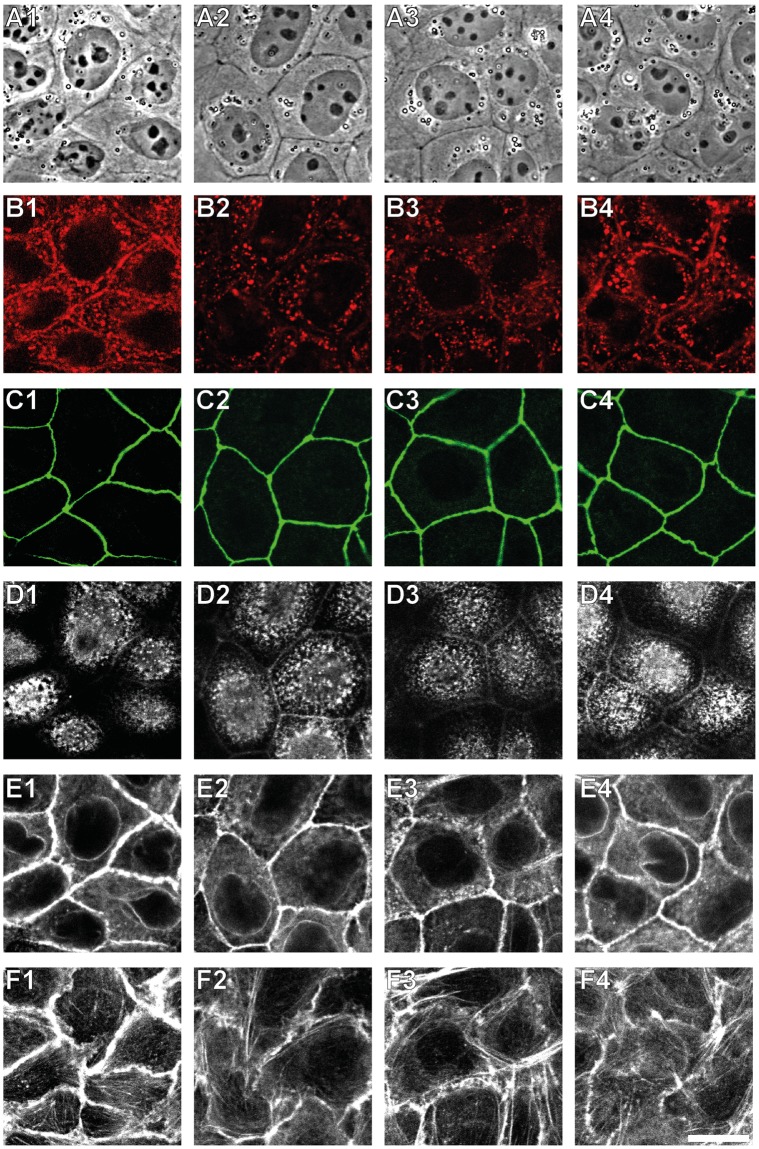
DTT interferes with E-cadherin mediated cell-cell junctions. (**A**) Phase contrast images. (**B–F**) Confocal fluorescence images for E-cadherin (**B**), zonula occludens-1 (**C**) and F-actin (**D**–**F**). The focus was set to the apical (**D**) and basal (**F**) cell site and to the focal place corresponding to the E-cadherin channel (**E**). 1: untreated cells. 2: Cells after incubation with 10 mm DTT for 3 h. 3: Cells 3 h after DTT removal. 4: Cells 6 h after DTT removal. Scale bar: 15 µm.

Viewed from the apical side, adherens junctions are placed beneath the tight junctional complex. The transmembrane protein E-cadherin forms this intercellular connection by association of the E-cadherin’s extracellular EC1 domain of neighbouring MDCK II cells. It has been shown that intact disulfide bridges play a pivotal role for functional cell-cell adhesion of epithelial cells^27^. 1,4-dithiothreitol (DTT) is known to reduce disulfide bridges and is therefore suitable to selectively compromise functional adherens junctions. We used this chemical instead of siRNA or administration of antibodies since we could easily remove the agent to examine the reversibility of opening of adherens junctions. Moreover, this procedure was highly reproducible. Notably, it is known that DTT might also activate integrins and thereby generates side effects^28^. Since we are only interested in the apical cortex it is reasonable to assume that contributions from adherens junctions dominate for the mechanical investigation. Confluent MDCK II cells were exposed to DTT for 3 h to examine the role of adherens junctions for tension regulation in epithelial cells. The cleavage of the E-cadherin mediated cell-cell connection by DTT is confirmed by fluorescence imaging after exposure of confluent MDCK II cells to 10 mm DTT for 3 h (Fig. 5). In control cells, E-cadherin is clearly enriched at the cell-cell boundaries in the central focal plane (Fig. 5B1), while after DTT treatment for 3 h the fluorescence signal for E-cadherin is spread within the cells (Fig. 5B2). After 3 h of exposure, DTT was removed from the cells and they were allowed to recover for 3 h (Fig. 5 column 3). Within this time interval no visible changes in the E-cadherin distribution are observed (Fig. 5B3). However, after a total recovery time of 6 h (Fig. 5 column 4), E-cadherin is again concentrated at the cell-cell boundaries indicating that the process is reversible (Fig. 5B4).

Phase contrast imaging shows no changes in the cellular structure during the entire observation period (Fig. 5 row A). In contrast to ZO-1 silenced cells, the cell monolayer stays intact after incubation with DTT-containing medium, also tight junctions are not affected by DTT treatment (Fig. 5 row C). The distribution of actin on the apical cell side remains unchanged (Fig. 5 row D), small point-like structures remain. Interestingly, in the central focal plane (Fig. 5 row E) (at the same *z*-position, where E-cadherin imaging is carried out) the actin fence, found as a solid ring in control cells (Fig. 5E1), is perturbed after 3 h of DTT treatment (Fig. 5E2). 3 h after removal of the drug the cells do not restore the original actin distribution (Fig. 5E3). Only after keeping the cells free from DTT for altogether 6 h, the actin ring reshapes and appears as homogeneous as in untreated cells (Fig. 5E4). In contrast, stress fibre formation at the basal cell side is not significantly affected by disruption of the adherens junctions (Fig. 5 row F).

**Figure 6:**
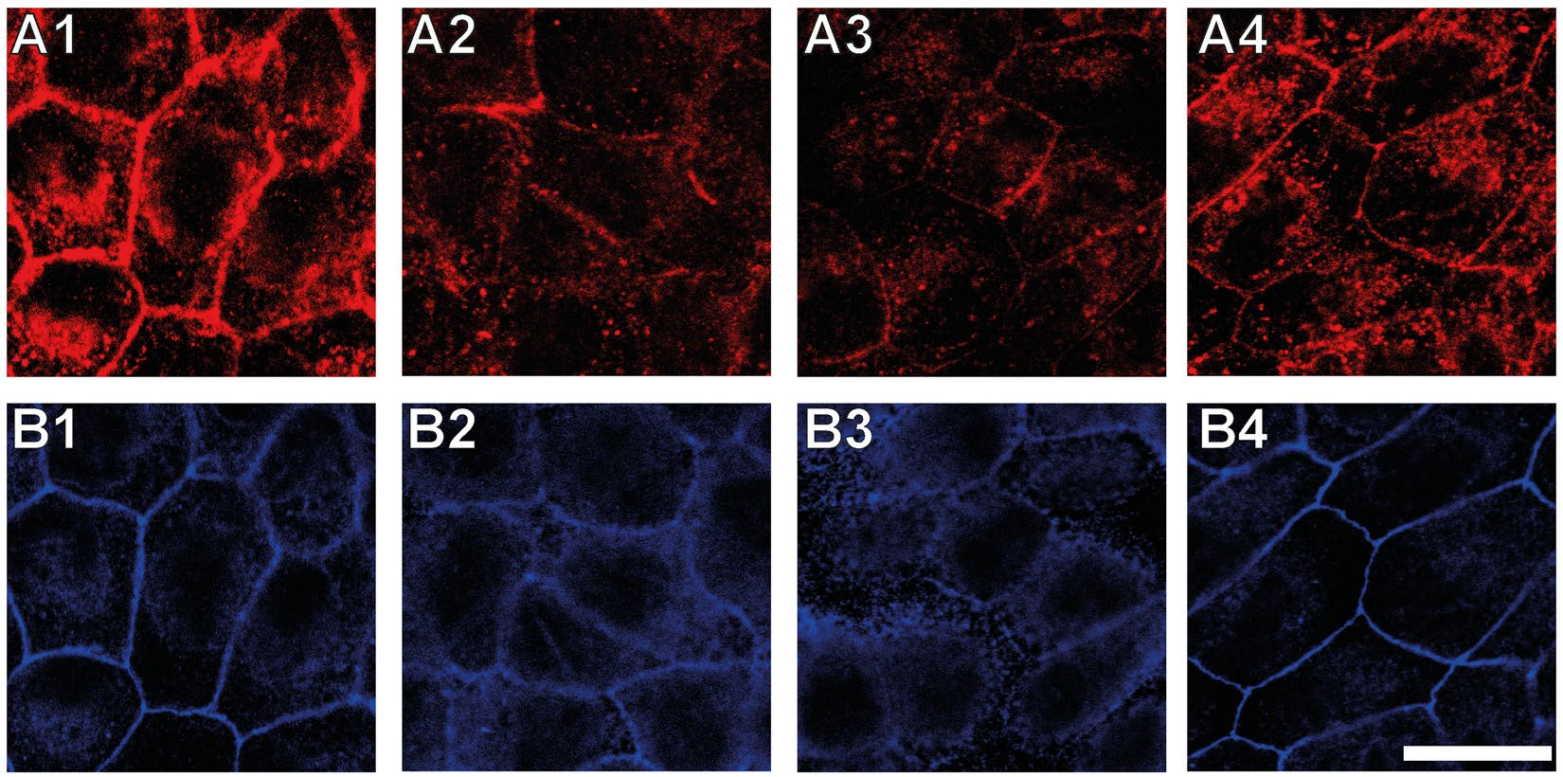
DTT effects the *β*-catenin distribution of epithelial cells. Fluorescence images for E-cadherin (**A**) und *β*-catenin (**B**), respectively. 1: Untreated MDCK II cells. 2: Cells after incubation with 10 mm DTT for 3 h. 3: Cells 3 h after DTT removal. 4: Cells 6 h after DTT removal. The images shown in (**A**) and (**B**) are recorded at the same focal plane. Scale bar: 20 µm.

The intracellular E-cadherin is known to be connected to the actin cytoskeleton via catenins and we found that the actin distribution at the lateral cell side is severely perturbed by DTT treatment (Fig. 5E1, E2). Therefore, we also investigated the distribution of *β*-catenin (Fig. 6). Control cells display a ring of *β*-catenin in the same focal plane, where E-cadherin is located (Fig. 6B1). After dissolution of the E-cadherin-E-cadherin bonds the *β*-catenin ring is also destroyed (Fig. 6A2, B2). The recovery of the *β*-catenin structure occurs within the same time as the E-cadherin reformation. 3 h after DTT removal the distributions of both, E-cadherin and *β*-catenin are still irregular (Fig. 6A3, B3). However, after keeping the cells free from DTT for 6 h, they finally manage to restore the distribution of E-cadherin (Fig. 6A4), *β*-catenin (Fig. 6B4) and F-actin (Fig. 5E4).

**Figure 7:**
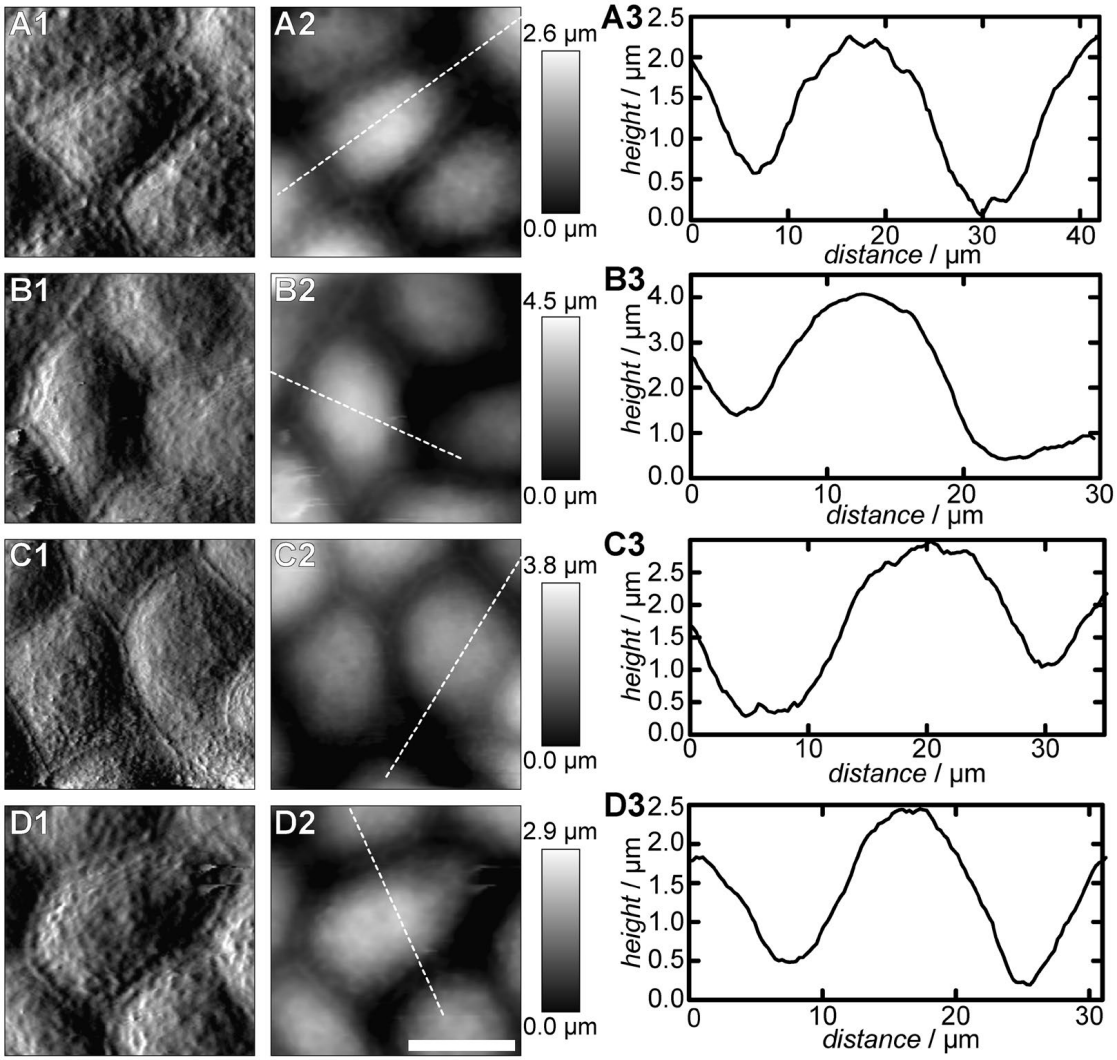
DTT treatment alters the morphology of MDCK II cells. (**A**) Untreated cells. (**B**) Cells imaged after treatment with 10 mm DTT for 3 h. (**C**) Cells after a recovery time of 3 h. (**D**) Cells 6 h after drug removal. 1: Deflection images. 2: Height images. 3: Height profile along the white dotted lines in 2. Scale bar: 15 µm.

AFM imaging confirms the presence of an intact cell monolayer during the whole experimental procedure (Fig. 7). However, a detailed inspection of the topography revealed that the morphology of the connected cells is altered by DTT incubation. Control cells display a typical height of the apical cap of $(2.0\pm 0.1)\,\mu {\rm{m}}$ (Fig. 7A). After exposure of the cells to 10 mm DTT for 3 h, a substantially elevated cap of $(2.8\pm 0.2)\,\mu {\rm{m}}$ is found (Fig. 7B). After removal of DTT and a recovery time of 3 h cells start to restore their initial topography (cap height: $(2.5\pm 0.2)\,\mu {\rm{m}}$, Fig. 7C). 6 h after DTT removal the height of the apical cap is completely restored to $(2.0\pm 0.2)\,\mu {\rm{m}}$ (Fig. 7D). Considering that the adherens junctions ‘outstretch’ the cells by pulling at adjacent cells, bulging of the cells after compromising the junctions and therefore removing the tension between them is expected and a strong hint to an altered cellular mechanics.

Next, we asked for the impact of DTT treatment and recovery from the treatment on the mechanical properties of the cells. Fundamental changes of the mechanical parameters are observed during the exposure to DTT and recovery from the drug treatment. Destroyed adherens junctions lead to a substantial drop in the overall tension from ${T}_{0}=(0.275\pm 0.010)\,\text{mN}/{\rm{m}}$ down to ${T}_{0}=(0.164\pm 0.005)\,\mathrm{mN}/{\rm{m}}$ after 3 h of DTT treatment. 3 h after DTT removal *T*_0_ is found to be $(0.315\pm 0.010)\,\mathrm{mN}/{\rm{m}}$, even higher than the initial value. Later, after a recovery time of 6 h the initial value is eventually restored (${T}_{0}=(0.265\pm 0.013)\,\mathrm{mN}/{\rm{m}}$) (Fig. 4A). DTT treatment also causes a halving of the apparent area compressibility modulus (${\hat{K}}_{{\rm{A}},{\rm{c}}{\rm{o}}{\rm{n}}{\rm{t}}{\rm{r}}{\rm{o}}{\rm{l}}}=(0.153\pm 0.008)\,{{\rm{n}}{\rm{N}}/\mu {\rm{m}}}^{3}$, ${\hat{K}}_{{\rm{A}},3{\rm{h}}{\rm{D}}{\rm{T}}{\rm{T}}}=(0.083\pm 0.006)$ ${\mathrm{nN}/\mu {\rm{m}}}^{{\rm{3}}}$. When the drug is removed, cells manage to restore this value within 3 h also with a slight overshoot. It is found to be ${\hat{K}}_{{\rm{A}}}=(0.194\pm 0.024)\,{\mathrm{nN}/\mu {\rm{m}}}^{{\rm{3}}}$. After 6 h of recovery from the DTT treatment, an apparent area compressibility modulus comparable to untreated cells is detected $({\hat{K}}_{{\rm{A}}}=(0.187\pm 0.078)\,{\mathrm{nN}/\mu {\rm{m}}}^{{\rm{3}}})$ (Fig. 4B).

DTT treated cells show a lowered membrane tension (${T}_{{\rm{t}}}=(0.121\pm 0.036)\,\mathrm{mN}/{\rm{m}}$) compared to untreated cells $({{\rm{T}}}_{{\rm{t}},{\rm{c}}{\rm{o}}{\rm{n}}{\rm{t}}{\rm{r}}{\rm{o}}{\rm{l}}}=(0.161\pm 0.003)\,{\rm{m}}{\rm{N}}/{\rm{m}})$. When the drug is removed for 3 h, *T*_t_ is found to be $(0.227\pm 0.010)\,\mathrm{mN}/{\rm{m}}$. After 6 h of recovery, a homeostasis occurs. The membrane tension drops again down to ${T}_{{\rm{t}}}=(0.188\pm 0.006)\,\mathrm{mN}/{\rm{m}}$ (Fig. 4C) close to the initial value.

In summary, we found that opening of adherens junctions by DTT leads to a considerable softening of the individual cells. Both, cortical and membrane tension drop and either the bending modulus of the composite shell is reduced or the excess membrane area is enlarged depending on the interpretation of the apparent area compressibility modulus. Interestingly, the cells entirely recover from this treatment after keeping them for 6 h in the absence of DTT. This holds true both for structure and mechanics. Cells reach their initial values by going through a state of higher stiffness as initially found for control cells (overshoot).

## Discussion

In the present study we examined how different cell-cell junctions contribute to the mechanical properties of individual epithelial cells and how the cells maintain tension homeostasis after selectively destroying the junctions.

It is clear that cell-cell junctions are important for providing efficient and selective barriers against the environment. This also includes a stable mechanical connection between adjacent cells that permits the cell layer to respond to external mechanical stress without losing its integrity. There are seminal studies addressing questions like, which forces are necessary within cells to establish strong cell-cell binding complexes^29,30^, how different types of junctions influence each other^31,32^, and how forces are transmitted within a cell layer^33–36^. However, it is still not entirely clear if and to what extent cell-cell junctions impact the mechanical behaviour of individual cells of a confluent monolayer.

When one of the major players of tight junctions, ZO-1, is depleted, we found that cells are visibly separated from each other. AFM imaging reveals that only a few finger-like connections between the cells remain (Fig. 2). Surprisingly, however, these significant alterations of the cell morphology have only a small effect on the mechanical behaviour of the individual cells that are still connected via adherens junctions. Slightly elevated overall tension and area compressibility moduli (Fig. 4) can be explained by an altered actin cytoskeleton (Fig. 3B2) with coarser structures on the apical side. This is in good agreement with results from Fanning *et al*. They also found larger aggregates of actin and accumulating myosin at the cell apex in ZO-1/-2 depleted MDCK II cells^11^. An enhanced myosin II level and higher actomyosin contractility may also contribute to higher tension values. Contrary to Fanning *et al*., however, we did not find a stronger actin belt at the lateral apical junctional complex region in response to ZO-1 depletion (Fig. 3C2). This may be due to the knock down of only ZO-1 and thereby explain the only moderately enhanced tension in our case. Compared to their work, we also found less pronounced stress fibre formation, when the cells are separated from each other (Fig. 3D2). Ikenouchi *et al*. demonstrated that polarisation of EpH4 epithelial cells is slowed down when ZO-1/-2 is lacking. Actin on the lateral cell side is separated between individual cells^37^. We found the same actin distribution in our experiments with MDCK II cells.

In our experiments cells undergo substantial morphological changes upon ZO-1 depletion (*vide supra*). The membrane-cytoskeleton attachment describing membrane tension *T*_t_, however, remains largely at the same level that we found for control cells. This supports the work from Fanning *et al*. who found that cell polarity and ezrin distribution is unaffected in ZO-1/-2 double knockdown cells.

In contrast to ZO-1 depletion, E-cadherin interference has dramatic consequences for the mechanical properties of MDCK II cells, while the impact on the morphology of the cell monolayer is small. Capaldo and Macara reported a swelling of the apical cap in E-cadherin silenced MDCK T23 cells^38^. Our AFM studies confirm this result, the removal of adherens junctions results in a substantial rounding of the cells indicative of an interfacial tension release. Considering that contractile cortical tension is balanced by the presence of cell-cell contacts it is expected that cells bulge into a more roundish shape after losing the cell-cell counter force. Interestingly, Capaldo and Macara report that *β*-catenin remains at the cell-cell-boundaries, whereas we found that the protein spreads within the whole cell after DTT treatment (Fig. 6B2). Changes in this protein distribution provoke a reduction of the overall tension and the area compressibility modulus since actin is no longer connected to the lateral cell membrane. A tension generating actin belt is partially degraded, as shown by confocal fluorescence microscopy at the central focal plane (Fig. 5E2). Studies on *Xenopus* revealed that cadherins influence the actin architecture^39^. Thus, the mechanical stability provided by the actin belt cannot be maintained after E-cadherin disruption. Additionally, we were able to show that recovery of the original E-cadherin distribution after DTT removal is accompanied by a recovery of the initial cell topography (Fig. 7). Interestingly, after only 3 h of recovery both mechanical parameters, ${T}_{0}$ and ${\hat{K}}_{{\rm{A}}}$, return to even higher values as found for untreated cells, which might be due to an altered geometry. At the same time *β*-catenin is still distributed within the whole cell interior. This is a strong hint that other regulatory mechanisms can rescue the mechanical integrity without participation of *β*-catenin. At this time point E-cadherin is already partly located at the cell-cell junctions. Other studies put the cadherin-catenin-actin complex as the only cadherin-cytoskeleton connection way into question^40^. However, there is evidence in the literature that force generation within the cell is necessary to establish cadherin mediated cell-cell contacts^41–43^. In our experiments cells start to rebuild adherens junctions after DTT removal through a temporary increase (overshoot) of the measured mechanical parameters, essentially forming stiffer cells than initially present. During the next 3 h keeping the cells in the absence of DTT, this effect is balanced out through a tug-of-war mechanism. Eventually the cells restore E-cadherin mediated cell-cell junctions (Figs 5B4 and 6A4). *β*-catenin is again concentrated at the E-cadherin enriched adherens junctions in accordance with junction formation described in textbooks^40^.

After AJ degradation we found a small decrease in membrane tension followed by an enhanced *T*_t_ level during E-cadherin reassembly. It is indisputable that membrane tension is largely governed by the attachment of the cytoskeleton to the plasma membrane^44^. In studies with *C*. *elegans* it was shown that the apical membrane-cytoskeleton linker ezrin homologue ERM-1 is necessary for apical junction formation^45^. In order to re-establish adherens junctions after DTT removal MDCK II cells might therefore enhance their ezrin level. This linker protein is then available for enhancement of the apical membrane-cytoskeleton connection leading to higher *T*_t_ values^26^. More pinning points to the membrane also increase the level of cross-linking of the actomyosin network that also allows the cells to adopt a higher cortical tension through motor activity. After 6 h, the cells eventually manage to return to a normal AJ formation reflected in a recovery of all three mechanical parameters (Fig. 4).

Tension homeostasis of epithelial cells depends on many complex mechanisms in single cells and the whole tissue. Our study shows that intact lateral cell junctions are not solely responsible for control of mechanical integrity. Tight junctions enable a sealed epithelial monolayer with a controlled perijunctional flux. The impact on mechanical stability of single cells of cell sheets is, however, small. The presence of intact tight junctions is synonymous with softer cells, maybe by decoupling the apical from the basolateral part of the polarised cell. In contrast, loss of functional adherens junctions results in a severe softening of cells that is, however, fully reversible.

## Material and Methods

### Cell culture

Madin-Darby canine kidney cells (strain II, MDCK II; Health Protection Agency, Salisbury, UK) were maintained in minimum essential medium (MEM) with Earle’s salts and 2.2 g/L NaHCO_3_ supplemented with 4 mm l-glutamine and 10% foetal calf serum at 37 °C in a 5% CO_2_ humidified incubator. Cells were grown to 70–90% confluency, released from culture flasks using trypsin/EDTA (0.05%/0.02%) and subcultured three times a week. Medium additionally contained penicillin (0.2 mg/mL), streptomycin (0.2 mg/mL), Amphotericin B (0.5 mg/mL) and HEPES (15 μm) during experimentation.

### Disruption of adherens junctions

Cells were seeded on Petri dishes (μ-Dish 35 mm, low Grid-500; ibidi, Martinsried, Germany) and grown to confluency. 1,4-dithiothreitol was dissolved in antibiotics and fungicide containing cell culture medium with a drug concentration of 10 mm. Cells were incubated with the drug containing medium for the indicated time at 37 °C.

### ZO-1 silencing

MDCK II cells were seeded on Petri dishes (μ-Dish 35 mm, low Grid-500; ibidi, Martinsried, Germany) and grown to 50% confluence. Pooled siRNA targeting zonula occludens-1 sequences GCAAAGACAUUGAUAGAAA, GAGAAGAAGUGACCAUAUU, CAAAAGAUCUGCAUCCUUA, and CCUGAACCAUGGUCGGAAA (siGENOME SMARTpool human TJP1 siRNA; Thermo Fisher Scientific, Lafayette, USA) were transfected using Lipofectamine® RNAiMAX transfection reagent (Life Technologies, Carlsbad, USA) according to the manufacturer’s instructions. Experiments were performed 72 h after incubation with siRNA.

For control measurements non-targeting siRNA sequences UAAGGCUAUGAAGAGAUAC, AUGUAUUGGCCUGUAUUAG, AUGAACGUGAAUUGCUCAA, UGGUUUACAUGUCGACUAA (siGENOME non-targeting siRNA Pool #2, GE Healthcare, Lafayette, USA) were applied.

### Cell labelling

Cells were grown on Petri dishes (μ-Dish 35 mm, low Grid-500; ibidi, Martinsried, Germany), treated as desired, and fixed with 4% paraformaldehyde in phosphate buffered saline without calcium and magnesium (PBS^−^) for 20 min. To block unspecific binding sites and to permeabilise the plasma membrane, cells were treated with blocking buffer (5% (*w*/*v*) bovine serum albumin (BSA), 0.3% (*v*/*v*) Triton X-100 in PBS^−^) for 30 min. For ZO-1 labelling the fluorescently labelled primary antibody (ZO-1 Monoclonal Antibody (ZO1-1A12), Alexa Fluor 488; Thermo Fisher Scientific, Schwerte, Germany) was diluted with dilution buffer (1% (*w*/*v*) BSA, 0.3% (*v*/*v*) Triton X-100 in PBS^−^) to a concentration of 5 μg/mL, and cells were incubated for 1 h at room temperature. E-cadherin was labelled by incubation of the permeabilised cells with a fluorescently labelled antibody (Alexa Fluor 488 or Alexa Fluor 555 Mouse anti-E-Cadherin (Clone 36), *c* = 5 µg/mL; BD Biosciences, Heidelberg, Germany). *β*-catenin staining was performed incubating permeabilised cells with mouse anti-*β*-catenin antibody (*c* = 5 µg/mL in dilution buffer; BD Biosciences, Heidelberg, Germany) for 1 h. The secondary antibody (Alexa Fluor 546-conjugated goat anti-mouse IgG; Life Technologies, Carlsbad, USA) was diluted with dilution buffer down to a concentration of 5 μg/mL and incubated with the cells for 45 min. F-actin was labelled by incubating cells with 165 nm Alexa Fluor 546-phalloidin or Alexa Fluor 647-phalloidin (Life Technologies, Carlsbad, USA) for 45 min. Between every labelling step, cells were rinsed three times with PBS^−^ for 5 min each on a vibratory plate (75 rpm).

Fluorescence imaging was carried out using a confocal laser scanning microscope (CLSM; FluoView1200; Olympus, Tokyo, Japan) using an 100x oil immersion objective (UPLFLN100xO2PH, NA = 1.3; Olympus, Tokyo, Japan). Fluorescence images are shown in false colours.

### AFM imaging

Cells were prepared as desired and fixed by incubation with 2.5% (*v*/*v*) glutaraldehyde in PBS^−^ for 20 min. Cell imaging was carried out using a Nanowizard® II, III or 4 atomic force microscope (JPK Instruments, Berlin, Germany) equipped with silicon nitride cantilevers (MLCT, *k* = 10 mN/m, Bruker AFM Probes, Camarillo, USA). The AFM was mounted on an inverse optical microscope (IX 81 or IX83, Olympus, Tokyo, Japan) to enable phase contrast microscopy during imaging. Cells were imaged in contact mode in PBS^−^ at room temperature with a scan rate of 0.5 Hz. Image processing was performed using software provided by the AFM manufacturer.

### AFM force indentation experiments

Force indentation cycles were recorded on the above-mentioned setup. Cantilevers (MLCT, *k* = 10 mN/m, Bruker AFM Probes, Camarillo, USA) were plasma cleaned (Argon, 30 s) and incubated with FITC-conjugated Concanavalin A (*c* = 2.5 mg/mL in PBS^−^; Sigma-Aldrich, Steinheim, Germany) for 1.5 h. This enables strong interaction between the cantilever and the plasma membrane during indentation and results in pulling out membrane tethers upon retraction of the indenter. The exact spring constant of each cantilever was determined using the thermal noise method^46^. Petri dishes containing samples of living cells were mounted on an inverted microscope and kept at 37 °C during experimentation. Cells were indented up to a force of 1 nN. After a dwell time of 0.5 s the indenter was retracted from the plasma membrane and membrane nanotubes were frequently pulled out of the plasma membrane. The indentation and retraction speed was set to 2 µm/s. These force indentation experiments were performed while scanning laterally across the sample, referred to as force mapping^25^.

### Data analysis of force indentation and retraction experiments

Force indentation curves were analysed as described in our previous studies^34^. Briefly, Force (*F*)-indentation (*δ*) curves were fitted by a polynomial starting at the contact point, where the indenter touches the cell membrane:

$$F(\delta )=a\cdot \delta +b\cdot {\delta }^{3}$$

Prefactors *a* and *b* were used to calculate the overall tension *T*_0_ and apparent area compressibility modulus ${\hat{K}}_{{\rm{A}}}$ taking the geometrical properties of the indenter with the half-opening angle $\varphi$ into account:

$${T}_{0}=\frac{a}{2\pi \,\cos ({\rm{\Theta }})},$$

and

$${\hat{K}}_{{\rm{A}}}=\frac{b}{2{\pi }^{2}(1-\,\cos ({\rm{\Theta }}))}\,\tan ({\rm{\Theta }}),$$

where $${\rm{\Theta }}=\frac{\pi }{2}-\varphi $$.

The advantage of this simplified model over more sophisticated ones, where the geometry of the cell is considered^47^, is its analytical tractability and that it only considers the area increase due to the presence of the indenter. It has been shown by Discher and co-workers that a polynomial fit (linear and cubic term) serves well to describe the experimental data^48^.

Membrane tension *T*_t_ was calculated from force steps (*F*_tether_) according to:

$${T}_{{\rm{t}}}=\frac{{F}_{{\rm{tether}}}^{2}}{8{\pi }^{2}\kappa }$$

$$\kappa =2.7\cdot {10}^{-27}{\rm{J}}$$ was chosen^25,49,50^.

An example of a force indentation and retraction cycle and the fitting procedure is shown in the supplementary information (suppl. Fig. S2).

## Electronic supplementary material

Supplementary Figures

## Data Availability

The datasets generated and analysed during the current study are available from the corresponding author on reasonable request.
