# Supplementary material for: Importance of integrity of cell-cell junctions for the mechanics of confluent MDCK II cells
Source: Sci Rep. 2018 Sep 20;8:14117. doi: 10.1038/s41598-018-32421-2 (PMC6148251; doi:10.1038/s41598-018-32421-2)
Supplement: Supplementary file 1 — Supplementary Figures [file 41598_2018_32421_MOESM1_ESM.pdf]

Importance of integrity of cell-cell-junctions for the mechanics of confluent MDCK II cells

Bastian Rouven Brückner[1], Andreas Janshoff[1]*

[1]University of Goettingen, Institute of Physical Chemistry, Tammannstr. 6, 37077 Goettingen, Germany

*correspondence: ajansho@gwdg.de,
phone: +49 551 39 10633, fax: +49 551 39 14411

Supplementary Figures

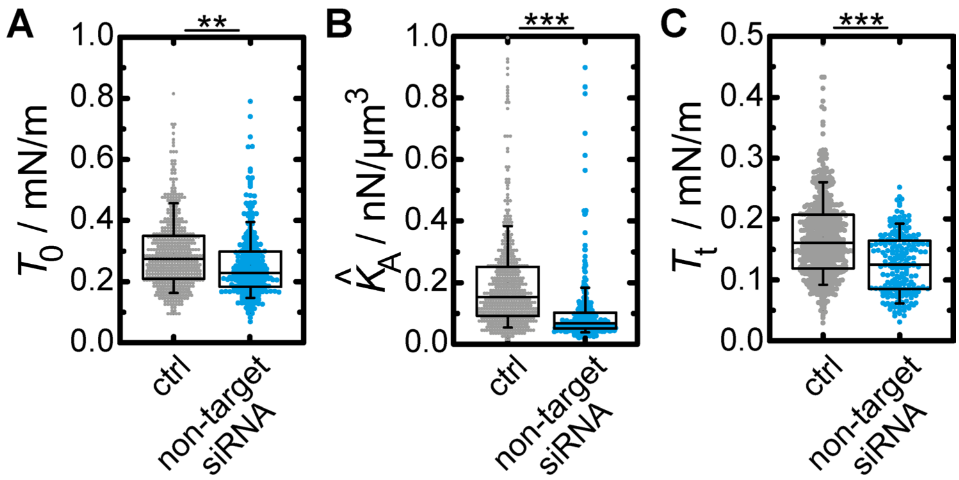

Figure S1| Control measurements of MDCK II cells using non-targeting siRNA. A: Overall tension T_0. B: Apparent area compressibility modulus $\hat{K}_A$. C: Membrane tension T_t. Box plots extend from the 25th to 75th percentile, whiskers from the 10th to the 90th. Grey dots show the same data as in the main text, extracted from force-indentation curves recorded on untreated cells (ctrl), light blue ones were recorded on cells exposed to non-targeting siRNA using the same transfection procedure as for ZO-1 silencing. The light blue data were originally published in Ref. [1]. For this publication we processed the data again applying the mechanical model described in the main text. A-B: n = 501 (ctrl), 327 (non-target siRNA) analysed force-indentation curves. C: n = 618 (ctrl), 218 (non-target siRNA) analysed force-retraction curves. A rank-sum test was performed to test the null hypothesis that the data of the indicated datasets are from populations with equal medians. Asterisks indicate that the null hypothesis was rejected at the 0.5% (**), or 0.05% (***) significance level.

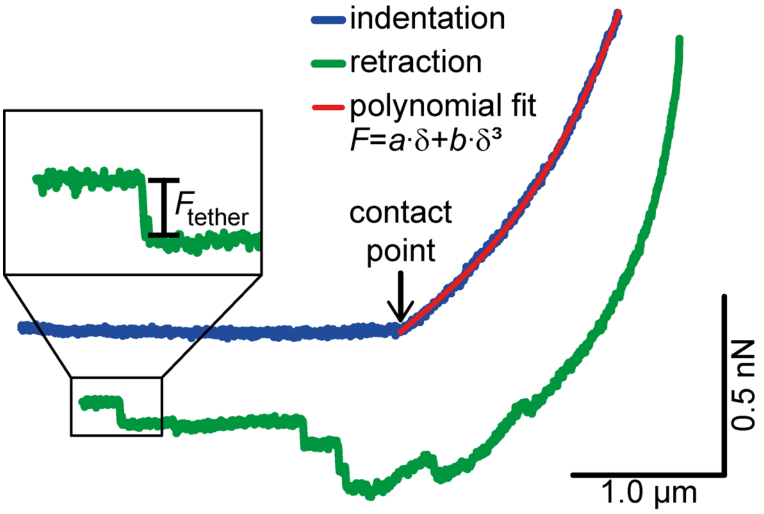

Figure S2| Analysation of AFM force indentation experiments. The force (F) indentation (δ) curve (blue) was fitted by a polynomial $F(\delta) = a \cdot \delta + b \cdot \delta^3$ starting at the contact point. Prefactors a and b were used to calculate the overall tension T_0 and the apparent area compressibility modulus $\hat{K}_A$ as described in the main text and in Ref. [2]. The tether rupture force extracted from the force retraction curve (green) was used to calculate the membrane tension T_t as described in the main text.

1 Brückner, B. R., Pietuch, A., Nehls, S., Rother, J. & Janshoff, A. Ezrin is a Major Regulator of Membrane Tension in Epithelial Cells. *Sci. Rep.* **5**, 14700 (2015).

2 Karsch, S., Kong, D., Großhans, J. & Janshoff, A. Single-Cell Defects Cause a Long-Range Mechanical Response in a Confluent Epithelial Cell Layer. *Biophys. J.* **113**, 2601-2608 (2017).
